# Critical assessment of alignment procedures for LC-MS proteomics and metabolomics measurements

**DOI:** 10.1186/1471-2105-9-375

**Published:** 2008-09-15

**Authors:** Eva Lange, Ralf Tautenhahn, Steffen Neumann, Clemens Gröpl

**Affiliations:** 1 Beatson Institute for Cancer Research, Proteomics and Mass Spectrometry Group, Scotland, UK; 2 Leibniz Institute of Plant Biochemistry, Bioinformatics and Mass Spectrometry, Halle, Germany; 3 Free University Berlin, Department of Mathematics and Computer Science, Berlin, Germany

## Abstract

**Background:**

Liquid chromatography coupled to mass spectrometry (LC-MS) has become a prominent tool for the analysis of complex proteomics and metabolomics samples. In many applications multiple LC-MS measurements need to be compared, e. g. to improve reliability or to combine results from different samples in a statistical comparative analysis. As in all physical experiments, LC-MS data are affected by uncertainties, and variability of retention time is encountered in all data sets. It is therefore necessary to estimate and correct the underlying distortions of the retention time axis to search for corresponding compounds in different samples. To this end, a variety of so-called *LC-MS map alignment algorithms *have been developed during the last four years. Most of these approaches are well documented, but they are usually evaluated on very specific samples only. So far, no publication has been assessing different alignment algorithms using a standard LC-MS sample along with commonly used quality criteria.

**Results:**

We propose two LC-MS proteomics as well as two LC-MS metabolomics data sets that represent typical alignment scenarios. Furthermore, we introduce a new quality measure for the evaluation of LC-MS alignment algorithms. Using the four data sets to compare six freely available alignment algorithms proposed for the alignment of metabolomics and proteomics LC-MS measurements, we found significant differences with respect to alignment quality, running time, and usability in general.

**Conclusion:**

The multitude of available alignment methods necessitates the generation of standard data sets and quality measures that allow users as well as developers to benchmark and compare their map alignment tools on a fair basis. Our study represents a first step in this direction. Currently, the installation and evaluation of the "correct" parameter settings can be quite a time-consuming task, and the success of a particular method is still highly dependent on the experience of the user. Therefore, we propose to continue and extend this type of study to a community-wide competition. All data as well as our evaluation scripts are available at .

## 1 Background

Mass spectrometry (MS) has become the predominant technology for both proteomics and metabolomics experiments. In shotgun proteomics, proteins are first digested, then the resulting peptides are separated by liquid chromatography. The fractions of the mixture are transferred to the mass spectrometer. Soft ionization techniques like matrix-assisted laser desorption ionization (MALDI) or electrospray ionization (ESI) and high resolving mass analyzers are used to identify the individual compounds by peptide mass fingerprinting (PMF) or by tandem mass spectrometry. The latter uses another step of fragmentation and MS analysis (MS/MS). Multiple technologies also exist in metabolomics applications, where mass spectrometers are coupled to gas chromatography (GC), liquid chromatography (LC) or capillary electrophoresis (CE) for separation. For recent reviews see [1,2]. In this paper our focus is on LC-MS in proteomics and metabolomics applications.

The quantitative information in a proteomics LC-MS map can be used in numerous applications [3,4] ranging from additive series in analytical chemistry [5], analysis of time series in expression experiments [6,7], to applications in clinical diagnostics [8], where statistically significant markers detect certain states of diseases. Common applications in metabolomics are: The verification of substantial equivalence [9], or the profiling of, e.g., biosynthetic mutants to reveal cross-talk between pathways [10]. What applications have in common is that the same components in different measurements have to be related to each other. As with every laboratory experiment, chromatographic separation is stable and reproducible only to a certain extent. The retention time often shows large shifts, and distortions can be observed when different runs are compared. Even the m/z dimension might show (typically smaller) deviations. The overall change in RT and m/z is called *warp*. Pressure fluctuations, or changes in column temperature or mobile phase result in distorted elution patterns, and can even cause changes in the elution order of components. Elution order changes are not unlikely if their retention times are similar [11]. For example, in one of our data sets the ground truth contained 88 verified matching peptide signals, but no more than 66 of them can be aligned without elution order changes (see last figure in additional File 1 for further information). The correction of the shift in RT and m/z is called *dewarping *according to the time warping problem of Sakoe and Chiba [12] in speech processing. The advent of high-throughput quantitative proteomics and metabolomics makes an efficient solution to this problem an important task.

In general, the data processing pipeline for label-free LC/MS data proteomics and metabolomics applications can be divided into the following steps:

1. Signal preprocessing and centroidization,

2. Detection and extraction of two-dimensional signals, so-called *features*, which are caused by chemical entities,

3. Intensity normalization,

4. Compensation of retention time distortions by dewarping,

5. Computation of a consensus map by assigning corresponding features across multiple maps,

6. Statistical analysis, feature identification, and the biological interpretation.

A typical label-free quantification protocol might be the connection of the proposed analysis steps, but it can also consist of the comparison of LC-MS maps on the raw data level [13]. The comparison of LC-MS raw maps enables the search for differentially expressed peptides directly by using multiway data analysis methods (e.g., PARAFAC [14]). Hence, a typical analysis pipeline for this approach avoids the steps 2 and 5, and merely includes the preprocessing and intensity normalization of the LC-MS raw maps, the correction of the retention time distortion, as well as the statistical analysis, feature identification and the biological interpretation of the data. We call the dewarping and thereby superposition of multiple LC-MS raw maps the *LC-MS raw map alignment problem*. Several algorithms have been designed to deal with this problem [15-19]. They avoid errors introduced by centroidization and feature finding algorithms, but they tend to have high runtimes and are liable to time order changes. Moreover, the algorithms are usually described for pairwise alignment and do not easily generalize to a multiple alignment of *N *maps. In this paper we will concentrate on the typical label-free quantification analysis pipeline and focus on the so-called *LC-MS feature map alignment problem*, which comprises the dewarping of multiple feature maps as well as the grouping of corresponding features in different maps. Since feature maps have a much smaller data amount than raw maps, they allow for much faster dewarping algorithms. On the other hand, signal preprocessing, centroidization and feature finding may also introduce errors. Therefore, the quality of the feature maps strongly depends on the reliability of these processing steps.

Within the last four years several algorithms for LC-MS feature map alignment have been developed [20-27]. These tools are either standalone tools or part of a whole framework for the analysis of MS based data. In this paper we concentrate on the comparison of the freely available feature map alignment algorithms implemented within the frameworks msInspect [25], MZmine [21], OpenMS [28] and XCMS [26], as well as the tools SpecArray [22] and XAlign [23] (see Table 1). Except for the alignment algorithm of MZmine, all methods estimate a linear (OpenMS, XAlign) or non-linear shift to correct the distortion of the RT dimension in all feature maps. The assignment of corresponding features and the determination of the consensus map is either done consecutively by processing the maps in a star-wise manner (MZmine, OpenMS, SpecArray, XAlign), or by a clustering approach (msInspect, XCMS). All algorithms take advantage of the more precisely measured m/z dimension to group corresponding features and to estimate the underlying warping function in RT. The general approach of the six different alignment methods compared by us will be described in the next section.

**Table 1:** Overview of alignment tools

**framework** *tool name*	input format	version	URL	programming language	operating system	source code available	modularity
**msInspect** *peptideMatch*	feature data in own tab- separated format	1.0.1		Java, R	Windows Linux MaxOS	✓	✓
**MZmine**	raw data	0.60		Java	Windows Linux MacOS	✓	-
**OpenMS** *MapAlignment*	feature data in featureXML or raw or peak data in mzData format	1.0		C++	Linux MacOS (Windows)	✓	✓
**SpecArray** *PepMatch, PepArray*	feature data in own binary format	2.1		C	Linux	✓	✓
**XAlign**	feature data in own tabular separated format	03.09.2007	request from the author	C++	Windows	-	✓
**XCMS**	raw data	1.10.7		R, C	Windows Linux MacOS	✓	✓

With the recent advent of LC-MS alignment reviews [13,29] it became obvious that a comprehensive unbiased performance study on a common benchmark set is needed to foster further competition and collaboration between the developers. In related fields, the Critical Assessment of Methods for Protein Structure Prediction (CASP) contests [30] and the Affycomp II Benchmark for Affymetrix GeneChip Expression Measures [31] have been quite fruitful in this respect. We have collected benchmark data sets from both proteomics and metabolomics experiments to compare *only the feature map alignment modules *of different software packages. We aim to minimize the influence of the preceding and subsequent processing steps. Therefore, we eliminated the influence of the individual signal processing modules by importing a common feature list. We furthermore abandoned the search for features in individual files based on features found in other measurements which is sometimes referred to as filling-in missing features. For proteomics, we have selected two data sets from the Open Proteomics Database [32], which have been used previously for the evaluation of the raw map alignment algorithm OBI-Warp [17]. For metabolomics data, no such public data repository currently exists, so we used two of our own data sets from a typical comparative metabolomics study. We are making these data sets available at .

The remainder of this paper is structured as follows: In Sections 2.1 and 2.2 the benchmark data sets and the definition of ground truth are described. Section 2.3 introduces the MS software packages and how they were configured for the benchmark. The evaluation criteria are defined in Section 2.4. The results of our comparison are presented in Section 3, followed by a discussion of the merits of the underlying algorithms, and a conclusion of expected future developments in Section 4.

## 2 Methods

Before we describe the experimental setup and signal processing for the evaluation data sets we introduce some definitions that are used throughout the following sections. In our context, a *feature *is the two-dimensional (RT and m/z) signal caused by a single charge variant of a chemical entity. Feature detection involves identifying the signal region in the raw data (usually a union of convex sets) and fitting a theoretical model (e. g. elution profile, isotope distribution) to the observed data. The map alignment problem has two aspects: (1) finding a suitable *transformation *of retention times, so that corresponding features will be mapped to nearby retention times, and (2) reporting the actual *groups *of corresponding features across multiple LC-MS feature maps. We will refer to these groups as *consensus features*, emphasizing that the individual features constituting a consensus feature should represent the same charge state of the same ionized compound. Referring to the consensus feature as a whole, one can then speak of an average retention time, mass charge ratio, etc. The collection of all consensus features constitutes a *consensus map*, which stores the correspondence information of all detected features in multiple LC-MS feature maps.

Ideally, each feature should be assigned to one consensus feature and each consensus feature should contain one feature from each map. However, limited dynamic range or large variation in the sample will lead to consensus features which do not extend across all LC-MS experiments. Artifacts of the feature detection phase, such as "broken" elution profiles, may also show up during the map alignment, resulting in consensus features which contain more than one feature from a particular map. As a special case, a consensus feature may consist of a single feature from a single map, if no other map contains the same charge state of the ionized compound. We will refer to these as *singletons*.

We consider the transformation of retention times as an intermediate step, because the downstream data analysis will mainly be concerned with groups of features and their average position, etc. rather than the distortions of retention times. The ultimate goal of multiple LC-MS feature map alignment is to derive a consensus map. This fact should be reflected by our quality metrics. An alignment method should create a "meaningful" partition of the feature maps: Corresponding features should be grouped in only one consensus feature instead of being split in multiple subsets, but the algorithm must also avoid grouping together unrelated features.

**Figure 1:**
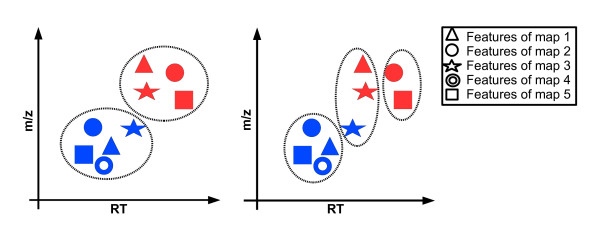
**Consensus precision and recall**. The left figure shows the two consensus features of a ground truth for the alignment of five feature maps. The features of the feature maps are distinguished by the five types of marker. Corresponding features in the different maps are illustrated by the same colour. The right figure shows three consensus features of a consensus map determined by an alignment algorithm. Note that the red features were assigned to separate consensus features, and the blue ones as well. The consensus feature in the middle even contains features from the same map. Thereby, the alignment results in a low recall value of (1/2)·(5/(2·5) + 4/(2·4)) = 0.5. Since most of the determined consensus features are "relevant" the method achieved a precision of (1/2)·(5/7 + 4/5) ≐ 0.76.

In Section 2.4 we introduce two measures that reflect the quality of a determined consensus map with respect to an optimal consensus map, the so-called *ground truth*. This is illustrated in Figure 1. The left part shows an optimal consensus map, representing the correspondence in four different feature maps. The right part shows a consensus map with various kinds of errors, which can occur in an alignment.

The quality of the transformation of retention times might also be assessed, but only after groups of corresponding features have been found. The transformation is often called a *warping function*, because original retention times *x *and transformed retention times *y *are related through a monotone increasing function *f*(*x*) = *y*. The difficulty with this approach is that the distance between corresponding features can be minimized by unrealistic, step-like warping functions. Hence in order to avoid overfitting, one has to include regularity (or "smoothing") conditions into the quality measure, which are hard to formalize.

In the following section we will describe the sample preparation of the complex biological proteomics and metabolomics data sets. Furthermore, we establish methods for the generation of proteomics and metabolomics ground truth consensus maps.

### 2.1 Proteomics data

We selected two proteomics data sets from the Open Proteomics Database (OPD) [32] resulting from two different experiments. The first data set originates from a dilution series of *Escherichia coli *and the other data set represents different cell states of *Mycobacterium smegmatis*. Both samples are of high complexity and provide typical alignment scenarios. They have previously been used for the evaluation of the LC-MS raw map alignment algorithm OBI-Warp [17].

We will briefly describe the sample preparation and the LC-LC-MS/MS analysis of the two experiments. Further information of the *E. coli *data set can be found on the OPD website and the *M. smegmatis *experiment is explicitly described in [33].

#### 2.1.1 Experimental setup

**Data set *P1***: LC-LC-ESI-IT-MS/MS

*E. coli *soluble protein extracts representing cells in exponential growth-phase were diluted in digestion buffer, denatured, and digested with trypsin. Tryptic peptide mixtures were separated by automated LC-LC-MS/MS. The injection quantity of the analyte was altered between two different runs: *021016*_*jp32A*_*10ul*_*3 *(10 *μL*, [OPD: opd00005_ECOLI]) and *021010_jp32A*_*15ul*_*1 *(15 *μL*, [OPD: opd00006 ECOLI]). We refer to these data sets as *P1*_*1 *and *P1*_*2*, respectively. Chromatography salt step fractions were eluted from a strong cation exchange column (SCX) with a continuous 5% acetonitrile background and 10-min salt bumps of 0, 20, 40, 60, 80, and 100 mM ammonium chloride. Each salt bump was eluted directly onto a reverse-phase *C*_18 _column and washed free of salt. Reverse-phase chromatography was run in and peptides were analyzed online with an ESI ion trap mass spectrometer (ThermoFinnigan Dexa XP Plus). In each MS spectrum, the three tallest individual peaks, corresponding to peptides, were fragmented by collision-induced dissociation (CID) with helium gas to produce MS/MS spectra. Centroided mzXML data and corresponding SEQUEST identification results of P1_1 and P1_2 were downloaded from the OPD.

**Data set *P2***: LC-LC-ESI-IT-MS/MS

*M. smegmatis *soluble protein extracts were diluted in digestion buffer, denatured, and digested with trypsin. Tryptic peptide mixtures were separated by automated LC-LC-MS/MS. The three different runs *6-17-03, 7-17-03*, and *6-06-03 *represent protein profiles of a *M. smegmatis *cell in middle exponential, early exponential and stationary phase [OPD: opd00009_MYCSM, opd00014_MYCSM, opd00028_MYCSM]. We refer to these data sets as *P2*_*1*, *P2*_*2*, and *P2*_*3*, respectively. The remaining setup is the same as above in *P1*. Centroided mzXML data and corresponding SEQUEST identification results of P2_1, P2_2, and P2_3 were downloaded from the OPD.

#### 2.1.2 Data extraction

The raw data had been exported in centroided mode by the instrument. Preprocessing and data extraction was performed using TOPP tools [34]. We converted all data from *mzXML *to *mzData *format using FileConverter and transformed the data into a uniformly spaced matrix by bilinear resampling using Resampler. The spacing of the transformed matrix was 1 Th and 1 second. Afterwards we detected and extracted peptide signals in the resampled raw data maps using FeatureFinder ignoring the charge states to provide fair means of comparison for all alignment tools. The sizes of the feature maps from the *P1 *and the *P2 *alignment test set are available as additional File 2.

#### 2.1.3 Ground truth

We established ground truth for the *P1 *and the *P2 *data sets by means of MS/MS information that was not available to the tested alignment procedures. As a consequence, our ground truth consist exclusively of features that can be annotated with a reliable peptide identification. This is discussed further below.

The reference method uses five steps: (1.) We establish an initial correspondence between MS/MS identifications and LC-MS features. (2.) We filter the peptide annotations based on the retention times of the features they are assigned to. The first two steps operate on each LC-MS/MS map individually. (3.) We compute an initial set of consensus features across multiple experiments. (4.) We reduce the list such that each feature is contained in at most one consensus feature. (5.) We filter the consensus features by comparing retention times across maps.

In the first step we scan through all peptide identifications. We disregard unreliable peptide identifications having a SEQUEST *XCorr *score less than 1.2. We check whether the RT and the m/z value of the precursor ion lies within the convex hull of a feature. In this case we assign the peptide identification to the feature. Each feature can be annotated with many peptide identifications originating from many MS/MS scans within the experiment. The values in parentheses in additional File 2 are the number of annotated features.

In the second step we filter the peptide annotations with respect to the retention times of the features they are assigned to. If a peptide identification is assigned to two features with very different RTs in one map, it is likely that one or both features are falsely annotated. This observation is used to filter out dubious identifications which otherwise might give rise to incorrect consensus features in the ground truth. For each peptide identification, we compute the mean *μ *and standard deviation *σ *of the RT positions of the features to which it is assigned. If *σ *> 100 s, then the identification is considered dubious and removed from all features. Moreover, the identification is removed from all features, if any, whose RT positions deviate by more than 2*σ *from *μ*. These filters are applied for each experiment separately.

In the third step we compute an initial list of consensus features, in which features with identical identifications are grouped across maps. In the previous steps we have computed a set of associations between peptide identifications from MS/MS and LC-MS features. The consensus features in our ground truth should have unique peptide identifications. Therefore we start by compiling a complete list of all peptide identifications over all experiments. Then we step through this list and for each identification we find the best-scoring features associated with it, but at most one from each experiment, and add these features to the corresponding consensus feature. In this way we maximize the sum of XCorr values for the peptide identifications in a consensus feature. We discard dubious consensus feature whose m/z standard deviation is greater than 1.

Let the *total XCorr *score of a consensus feature be defined as the sum of XCorr values of all features contained in it. After step three, it is possible that a feature is contained in different consensus features from the initial list. In the fourth step we reduce the initial list such that each feature is contained in at most one consensus feature, whose total score is the largest among all consensus features containing it. We have developed a simple "greedy" strategy to achieve this goal. The purified list of candidate consensus features is sorted in order of decreasing total score. In each step we extract a consensus feature with maximum total XCorr score from the list. This consensus feature is added to the consensus map, and all consensus features having a non-empty intersection with it are also removed from the list. The process is iterated until no more consensus features can be found, i. e., the list has become empty.

In the fifth step, we apply a final filter for outliers and dubious identifications by comparing retention times across maps. We calculate the RT sample variance within all consensus features in the consensus map and discard consensus features whose standard deviation is greater than 2 times the sample standard deviation. Since this filter relies upon RT information and hence bears the risk of introducing bias into the ground truth, we confirmed that the removed consensus features are indeed outliers by visual inspection.

The numbers of consensus features in the ground truth are also shown in additional File 2. A ground truth is only considered if its number of consensus features corresponds to a least 10% of the number of annotated featues in the aligned feature maps.

As stated above, the assignment used as a ground truth is restricted to features in different feature maps that were annotated by a peptide identification. We believe that this will not introduce a bias toward any of the tools, based on the assumption that the features, which are selected for MS/MS fragmentation are chosen randomly and independently with the same probability *p*. For simplicity, consider the case of pairwise alignment. The extension to multiple map alignment will be discussed in Section 2.4. The classical *precision *value is defined as TP/(TP + FP). Note that the denominator does not depend on the ground truth, and the enumerator is expected to be a constant fraction TP = *p *·TP* of the "real" true positive number TP*. Thus, it is still possible to *compare *the probability that a computed consensus feature is contained in the ground truth between the different tools, although the absolute precision values will be underestimated by a factor of *p *using the available ground truth. The *recall *value TP/(TP + FN) is not affected by such a bias, since both *TP *and *FN *will be underestimated by a factor of *p*, which cancels out. Hence, the classical recall value can still be used as an estimator for the probability that an "existing" consensus feature is actually computed by the tool.

### 2.2 Metabolomics data

We have selected a typical *Arabidopsis thaliana *metabolomics experiment, with different plant lines and treatments measured at multiple time points in triplicates. The same samples were measured on two different LC-MS setups as follows.

#### 2.2.1 Experimental setup

Preparation of Extracts

Freshly ground *Arabidopsis thaliana *leaf tissue (130 ± 5 mg) was subjected twice to the following extraction procedure: mixing with 200 *μL *of methanol/water, 4/1 (v/v), sonication at 22°C for 15 min and centrifugation for 10 min. Both extracts were combined and evaporated at reduce pressure in a vacuum centrifuge at ambient temperature. The remaining residue was redissolved in 400 *μL *methanol/water, 3/7 (v/v).

**Data set M1: **Capillary LC-ESI-QTOF-MS

1 *μ*l of the extract was separated using an Ultimate capillary LC system (Dionex) on a modified *C*_18 _column (GROMSIL ODS 4 HE, 0.3 × 150 mm, particle size 3 *μm*, Alltech-Grom) applying a binary acetonitrile-water gradient at a flow rate of 5 *μLmin*^-1^. Eluted compounds were detected from m/z 75 to 1000 by an API QSTAR Pulsar i (Applied Biosystems/MDS Sciex) equipped with an Ionspray electrospray ion source in positive ion mode. Accumulation time was 2 s. Mass resolution for [*M *+ *H*]^+ ^of a calibration peptide was RFWHM (resolution full width at half maximum) = 8500 at 829 m/z.

**Data set M2: **LC-ESI-QTOF-MS.

10 *μ*l of the *A. thaliana *extract were separated using a Agilent 1100 Series HPLC system on a modified *C*_18 _column (Atlantis dC18, 2.1 × 150 mm, particle size 3 *μm*, Waters) applying the same binary gradient as above at a flow rate of 200 *μLmin *^-1^. Eluted compounds were detected from m/z 100–1000 by a MicrOTOF-Q (Bruker Daltonics) equipped with an Apollo II electrospray ion source in positive ion mode. Accumulation time was 1.5 s. Mass resolution for [*M *+ *H*]^+ ^of a calibration peptide was RFWHM = 14000 at 829 m/z.

#### 2.2.2 Data extraction

All data were exported in centroid mode by the converter software from Applied Biosystems and Bruker, respectively. The feature finding was done using XCMS [26] using the parameters *method *= "*centWave*", *peakwidth *= *c*(20, 50), *snthresh *= 5, *ppm *= 120 for the data set M1 and *ppm *= 30 for the data set M2, respectively. The number of features for each file is available as additional File 3.

#### 2.2.3 Ground truth

In contrast to the proteomics data sets, usage of MS/MS information and SEQUEST annotation are not applicable. Compound spectra libraries exist for GC/EI-MS, but no extensive set of reference spectra is available for LC-ESI-MS. However, a relative annotation of "anonymous" substances is sufficient for the purpose of our alignment evaluation.

For soft ionization methods like LC-ESI-MS, different adducts (e.g. [*M *+ *K*]^+^, [*M *+ *Na*]^+^) and fragments (e.g., [*M *- *C*_3_*H*_9_*N*]^+^, [*M *+ *H *- *H*_2_0]^+^) occur. Using these known mass differences and verification techniques such as peak shape comparison by correlation analysis, features which originate from the same substance can be grouped together as annotated feature groups. Even if the substances are unknown, their spectra can be reconstructed in this way. Details are described in [35].

We used features that do not only have the same retention time but also show high correlation (Pearson correlation coefficient > 0.9) in their chromatographic peak shapes to create annotated feature groups. The correlation verified feature annotations were created using the R-Package *ESI*, which can be downloaded from .

Only those highly confident feature groups that were reproducible over at least four files and show limited deviation across the files (data set M1: ΔRT = 90 s, Δm/z = 0:02 Th, data set M2: ΔRT = 20 s, Δm/z = 0:01 Th) were used to create a verified alignment of these feature groups. Subsequently, the aligned feature groups were split up into their consensus features, which form the alignment ground truth. The number of features for each file and the size of the ground truth for each alignment are available in the additional File 3.

### 2.3 Computation of alignments

In the following subsections we will shortly describe the general approach of the six alignment methods as well as their most relevant parameters. Furthermore, we present our procedure to import the input feature lists into the different tools. Each program provides a consensus map in a proprietary file format which was parsed for the evaluation.

#### 2.3.1 OpenMS

The open source framework OpenMS [36] offers a multiple LC-MS map alignment algorithm [28] for raw as well as feature maps.

The maps are aligned in a star-wise manner with the most complete map as the reference map. The correction of the warp in RT and m/z and the determination of a consensus map are performed in two steps called *superposition phase *and *consensus phase*. This modularization allows for the implementation of a general algorithm that either aligns multiple raw maps using just the superposition phase, or aligns multiple feature maps applying both phases. In the superposition phase the parameters of a suitable affine transformation are determined using a general paradigm for point pattern matching algorithms called *pose clustering*. The optimal transformation, which is defined as the transformation that maps as many elements of one map as possible close to elements in the other map, is determined by a so-called *voting schema*. The pose-clustering algorithm considers the different measuring accuracies of the RT and m/z dimension as well as the intensity information of the LC-MS map elements. After the estimation of the initial transformation by the pose-clustering approach, landmarks are searched in the two maps. These landmarks are used for the refinement of the affine warp by a linear regression step. The following consensus phase is based on a nearest neighbors search and determines the final consensus map given the dewarped feature maps. The OpenMS multiple feature map alignment algorithm is implemented in the TOPP tool MapAlignment. The most important parameter for the user are *precision*_RT_, *precision*_m/z _and *mz*_*bucket*_*size*. The parameter *mz*_*bucket*_*size *is a parameter for the superposition phase. It restricts the computation of all possible transformations by mapping only features in both maps that have similar m/z positions. Whereas, *precision*_RT _and *precision*_m/z _are parameters of the consensus phase that define the maximal distance of corresponding features for the grouping process. The metabolomics feature lists were converted into the featureXML input format by the FileConverter TOPP tool.

#### 2.3.2 msInspect

The multiple feature map alignment algorithm presented in [25] is part of the open source LC-MS analysis platform *msInspect*. The software package is written in the platform independent language Java and is freely available at .

Before a consensus map, the so-called *peptide array*, is determined the algorithm corrects the non-linear distortions of the RT dimension of all maps in a star-wise manner with respect to a certain reference map. It is assumed that the distortion in RT is explained by a global linear trend plus a remaining non-linear component. In the first step, the linear trend is estimated using the most intense features with similar m/z positions. This initial model of the RT transformation is used to iteratively determine a non-linear transformation using smoothing-spline regression methods from the previous model. After dewarping all maps, a global alignment is performed by applying divisive clustering, with user-supplied tolerances in RT and m/z of assigned features. The algorithm optionally offers the automatic choice of the optimal RT and m/z tolerances using the quality of clustering. The quality of the alignment is defined by the number of clusters that include at most one feature from each map.

msInspect uses various tsv (tab-separated values) files for input and output. We implemented utilities for converting data from our feature map format featureXML into the msInspect tsv format and to extract the resulting consensus map from the msInspect output files. The alignment algorithm of msInspect provides the setting of two parameters: scanWindow, which is the maximum size of a consensus feature in time space, and massWindow, the maximum size of a consensus feature in mass space. The option – optimize is used to determine the best choices for the two parameters with respect to the number of *perfect matches*, which contain exactly one feature of each map. We used the parameters suggested by the optimizer but also different parameters to evaluate msInspect's alignment algorithm.

#### 2.3.3 SpecArray

Li et al. [22] developed a multiple feature map alignment algorithm embedded in the open source software suite *SpecArray *.

The proposed algorithm computes all pairwise alignments and combines them to a final consensus map. To correct the distortion in RT a retention time calibration curve (RTCC) is iteratively computed for each pairwise alignment by pairing features with similar m/z values to construct an original feature pairs set. The RTCC curve is estimated by minimizing the root mean square distance of the features' RT positions to the monotonic function. Pairs with a small pairing score are removed and the reduced set of feature pairs is again used to estimate a RTCC. The two steps are repeated until only the pairs with a high pairing score remain and each feature in one map is paired with at most one feature in the other map. The final RTCC curve and the distance of peptides in m/z is used to select likely and unique feature pairs from the original set of feature pairs. The combination of all pairwise alignments yields the final consensus map, or the so-called *super list*. The parameters for the alignment algorithm are hard-coded and cannot be changed by the user. Calculating all pairwise alignments results in a high runtime and makes the algorithm inapplicable for the comparison of a large number of feature maps. SpecArray provides two tools for the alignment of feature maps. Whereas, PepMatch performs the actual alignment step, PepArray can be used for the postprocessing and filtering of the consensus map. We avoid the filtering step and use the unprocessed final consensus map for evaluation purposes.

We implemented software to convert our feature map format featureXML into the SpecArray's binary feature format pepBof. Furthermore, we forced SpecArray to directly export our consensus format by the addition of some lines of code to the sources of PepMatch.

#### 2.3.4 XAlign

Zhang et al. [23] propose a stand-alone tool, called *XAlign*, for the alignment of multiple feature maps. The Xalign software for Windows is available upon request from the author.

*XAlign *computes in a first step a so-called gross-alignment, where the algorithm corrects a systematic shift in RT. In the second step, a final consensus map, the so-called *micro alignment*, is determined. The gross-alignment algorithm aligns multiple maps in a star-wise manner, where the reference map is chosen as follows: for all predefined RT and m/z windows the most intense features of each map are determined. If a window contains features from all maps, the features are called significant and their intensity weighted average mean RT position is calculated. The map with the minimal difference of all its significant features to the averaged RT positions is chosen as the reference map. Afterwards, all other maps are dewarped with respect to the reference by estimating a linear function that minimizes the mean absolute deviation of the RT positions of significant features. In the micro-alignment phase features yielding a high correlation coefficient are successively grouped together and establish the final consensus map. XAlign [23] is designed as a component of a data analysis pipeline for protein biomarker discovery. The stand-alone executable runs in the Windows command line. It reads tab-separated feature lists and generates several output files including the alignment table and peak statistics.

#### 2.3.5 XCMS

The XCMS package presented in [26] is part of Bioconductor [37], a larger open source software project for bioinformatics written in the platform-independent programming language R. All Bioconductor packages can be obtained from . XCMS is designed for both LC/MS and GC/MS data. It includes functionality for visualization, feature detection, non-linear retention time alignment and statistical methods to discover differentially expressed metabolites. We modified XCMS to skip the feature detection step and imported the featurelists directly from feature map format featureXML. XCMS' feature-matching algorithm makes use of fixed-interval bins (e.g., 0.1 Th wide) to match features in the mass domain. After this initial binning of features by mass, groups of features with different retention time in each bin are resolved. Kernel density estimation is used to calculate the distribution of features in chromatographic time and subsequently boundaries of regions where many features have similar retention times are identified.

XCMS supports an optional retention time correction step where "well-behaved" groups of features are used to calculate a nonlinear retention time deviation for each sample. The resulting deviation profiles are then used to correct the retention times of the original samples. The matching and retention time correction procedure can be repeated for an increasingly precise alignment. However, we observed that it is hard to predict whether the retention time correction will actually lead to a better consensus map and depends on the input. Therefore, we decided to report results both without and with the optional retention time correction step.

#### 2.3.6 MZmine

The MZmine toolbox [38] for processing and visualization of LC/MS data is used via a graphical user interface. Due to its implementation in Java it is platform independent. MZmine is open source and can be downloaded from . We modified MZmine to skip the feature detection step and import featurelists instead.

MZmine's alignment approach does not estimate any dewarping transformations. The toolbox currently implements a simple alignment method utilizing a so-called *master feature list*, where features from each map are aligned against the master list. A score function is used to compute the similarity of a feature and a row of the master list, which represents the current consensus feature. If the score obtained between the best matching master list row and a feature is "good enough" (both the m/z and retention time difference are within tolerances) the feature is assigned to that row, otherwise it is appended to the master list. MZmine offers two alignment algorithms, "slow aligner" and "fast aligner", which differ in the implementation of the score function. We found only minimal differences in the alignment quality of both algorithms so we used the "fast aligner" due to the better runtime.

#### 2.3.7 Parameters

We performed extensive test runs to optimize the parameters controlling the tolerance in RT and m/z for our test data. Using the known deviations of the data as a starting point we varied the parameters of each tool within reasonable ranges. The parameters which yielded the best results on the first experiment of each data set were choosen. The final settings are shown in Table 2.

**Table 2:** Alignment parameters

**Tool**	**Parameter**	**Metabolomics Data**		**Metabolomics Data**	
		Data Set P1	Data Set P2	Data Set M1	Data Set M2
msInspect	massWindow	1.5	1.5	0.1	0.05
	scanWindow	250	300	250	300
MZmine	m/z tolerance size	1.5	1.5	0.03	0.025
	RT tolerance size (absolute)	150	300	50	30
OpenMS	m/z bucket	0.5	0.5	0.1	0.01
	precision m/z	2	2	0.1	0.1
	precision RT	150	300	100	100
SpecArray	(hard coded parameters)	-	-	-	-
XAlign	m/z variation	2	2	0.04	0.03
	retention time variation	3	3	0.5	0.5
XCMS	mzwid	2.5	2.5	0.15	0.05
	bw	40	80	30	30
	retcor method	loess	linear	loess	loess
	span	0.75	-	0.75	0.75

### 2.4 Evaluation

The performance of an information retrieval system can be assessed using the *precision *and *recall *values. Our evaluation of the map alignment problem will follow these lines. As stated in the beginning of this section, the correction of retention times is a very important aspect of the LC-MS map alignment problem, and there is a trade-off between the smoothness of the warping function and the remaining distance among matched features. But at the end, the purpose of warping the retention times is to find groups of corresponding features that are reported as consensus features, which is why our analysis focuses on this aspect of the map alignment problem. That is, we will evaluate the quality of the *consensus map *rather than the *warping function*, because we consider the latter an intermediate step for the map alignment problem. Given a "query" feature in one map, the consensus map can serve to retrieve related "items" in the other maps. Consensus features are simply taken as sets of features; assigning an appropriate average position to these sets etc. is another problem and not addressed here.

In the frequentist interpretation, *precision *is the probability that a found item is relevant, whereas *recall *is the probability that a relevant item is found. In the special case of pairwise map alignment, the relevant items are matching features; an item is either found or not. In order to extend these concepts to the multiple map alignment problem, we need to deal with consensus features that do not contain features from all maps, as well as consensus features reported by tools, that overlap but are not identical to the ground truth.

Let us denote the consensus features in the ground truth by gt_*i*_, where the index *i *runs from 1 to *N*. Likewise, the consensus features from the tool will be denoted by tool_*j*_, for index *j *= 1,...,*M*. We consider the set of consensus features from the tool that contain at least two features (so that they can be used to retrieve items) and intersect with a given consensus feature from the ground truth. Thus, for each index *i *let us denote by *M*_*i *_the set of all indices *j *such that |tool_*j*_| ≥ 2 and |gt_*i *_∩ tool_*j*_| > 0. Now we can look at the cardinality of this index set, |*M*_*i*_|. In some way, this is the number of "parts" into which consensus feature gt_*i *_from the ground truth has been "split up" by the tool. But we can also look at the union of these consensus features, $\text{t}\tilde{\text{oo}}{\text{l}}_{i}:=\cup_{j\in M_{i}}{\text{tool}}_{j}$. Then $\text{t}\tilde{\text{oo}}{\text{l}}_{i}$ is the set of all items that can be retrieved if the query belongs to gt_*i*_.

Therefore, following the classical definition of precision and recall, we define the *alignment precision*:

$${\text{Precision}}_{\text{Align}}=\frac{1}{N}\sum_{i=1}^{N}\frac{\left|{\text{gt}}_{i}\cap \text{t}\tilde{\text{oo}}{\text{l}}_{i}\right|}{\left|\text{t}\tilde{\text{oo}}{\text{l}}_{i}\right|}$$

and the *alignment recall*:

$${\text{Recall}}_{\text{Align}}=\frac{1}{N}\sum_{i=1}^{N}\frac{\left|{\text{gt}}_{i}\cap \text{t}\tilde{\text{oo}}{\text{l}}_{i}\right|}{\left|M_{i}|\cdot |{\text{gt}}_{i}\right|}.$$

The factor |*M*_*i*_| in the denominator serves as a penalty for breaking up a consensus feature from the ground truth. Note that in the case of pairwise alignments, the summands in these definitions are either zero or one, and our definitions become equivalent to the classic precision and recall. Thus, their names are justified as generalizations. A perfect alignment will have both measures equal to one. False positives (erroneously grouped features) lower the alignment precision; false negatives (erroneously unaligned features) lower the alignment recall.

An example is shown and calculated in Figure 1.

An R script was written for the automated computation of the recall and precision values. The runtimes were measured as wall-clock time including all file input/output while no other programs were running. All measurements were done on an AMD Athlon 64 X2 Dual Core Processor 4800+ with 2 GB RAM running Linux (Ubuntu 6.06). Since XAlign does not run under Linux, we evaluated it under Windows XP running in a virtual machine using VMWare Workstation 5.5.3 on the same computer (native Windows XP should typically be 10–20% faster). The reported wall-clock runtimes are cumulative over all runs per data set.

## 3 Results

The proteomics (P1, P2) and metabolomics (M1, M2) data sets pose different challenges for the alignment tools. Each tool has to correct the global trend of the retention time variation resulting from the flow rate variability from experiment to experiment. Furthermore, it has to overcome local distortions resulting from e. g. gradient noise or temperature changes and assign corresponding features across the different maps.

**Figure 2:**
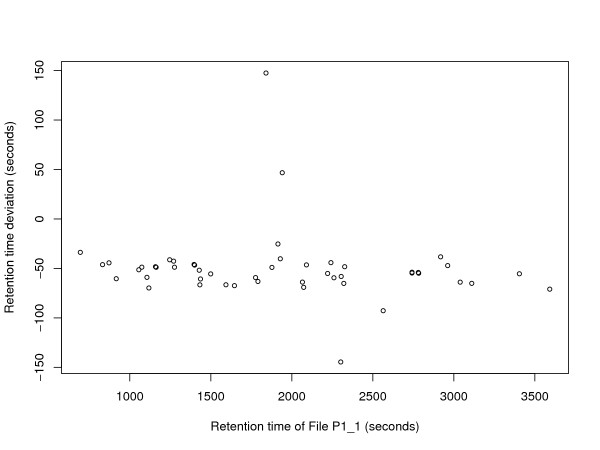
**Retention time deviations of data set P1**. Exemplary plot of retention time deviations in the ground truth of data set P1. Retention time deviation of File P1_2 is plotted against retention time of File P1_1 (fraction 100).

**Figure 3:**
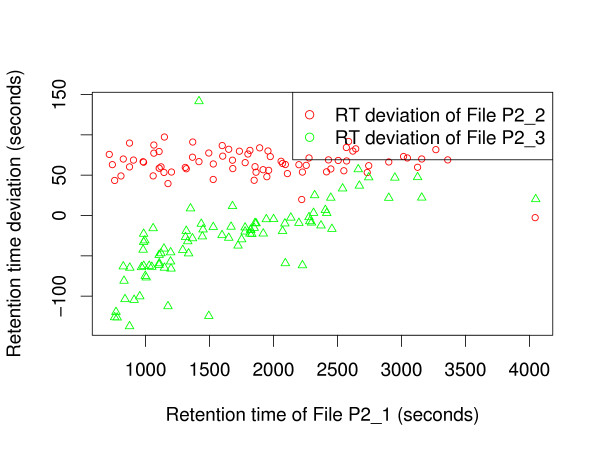
**Retention time deviations of data set P2**. Exemplary plot of retention time deviations in the ground truth of data set P2. Retention time deviations of File P2_2 and P2_3 are plotted against retention time of File P2_1 (fraction 20).

To illustrate the ground truth established for these data sets, we plot the retention time deviation versus the retention time. Figure 2 shows a significant shift between corresponding features in fraction 100 of P1_1 and P1_2, but almost no difference in scale. Figure 3 shows that fractions 20 of P2_2 and P2_3 are slightly scaled with respect to P2_1, but apart from that the retention times are in fact better correlated. While an average absolute retention time deviation of 57 s can be observed in the ground truth maps of P1, the average absolute retention time deviation for P2 is 131 s (before retention time correction). The retention time deviation plots for each single fraction of the data sets P1 and P2 are available as additional File 1.

**Figure 4:**
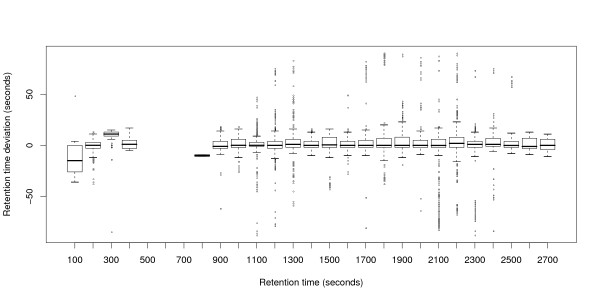
**Retention time deviations of data set M1**. Box-whiskers-plot showing the retention time deviations in the ground truth of data set M1.

**Figure 5:**
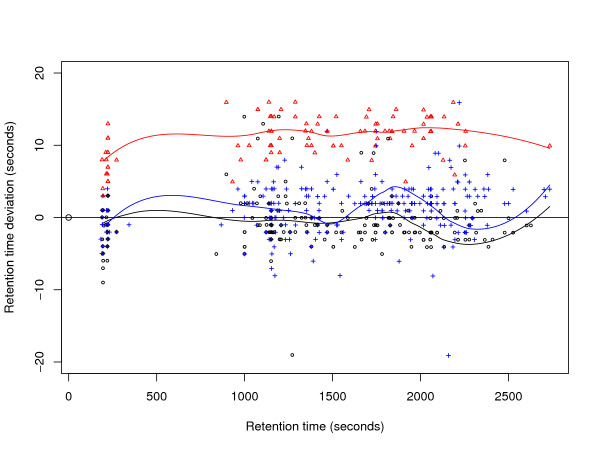
**Retention time deviations in the ground truth of three randomly chosen files from data set M1. **Loess regression curves were superimposed for better visualization.

**Figure 6:**
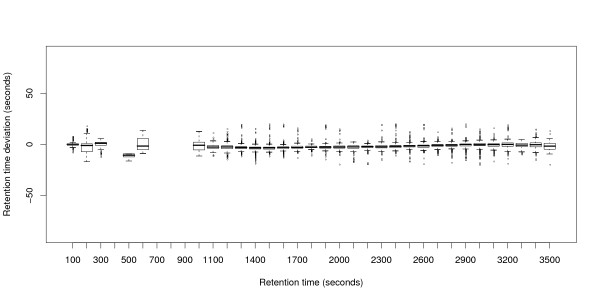
**Retention time deviations of data set M2**. Box-whiskers-plot showing the retention time deviations in the ground truth of data set M2.

**Figure 7:**
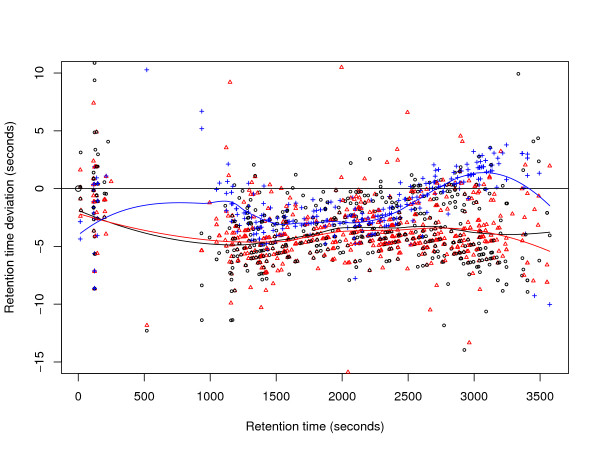
**Retention time deviations in the ground truth of three randomly chosen files from data set M2.** Loess regression curves were superimposed for better visualization.

The metabolomics data sets M1 and M2 contain a larger number of experiments (24 resp. 44). Therefore, we use box-whiskers plots for visualization. Figures 4 and 6 show that variation is higher in M1 than in M2, but still much smaller than in P1 or P2. The average absolute retention time deviation for the ground truth of the metabolomics data sets M1 and M2 is 5.4 s and 2.7 s respectively. Presumably, "large" deviations are the reason for most of the alignment errors. Loess regression curves for three randomly chosen files show that the global trends are not as pronounced as the local variation, see Figures 5 and 7.

Both proteomics data sets challenge the ability of the alignment tools to correct strong retention time variations. Especially the data of P2, which were measured during several weeks and show huge retention time deviations of around 13 minutes, confront the dewarping step of the tools with a serious problem. However, the highly complex metabolomics data sets reveal the capability of the alignment tools to assign the correct features across multiple maps. The maximum retention time deviations of feature maps in M1 and M2 are only 90 s and 20 s respectively, without an obvious global trend. The warps are mainly affected by local non-linear distortions of retention times similar to uncorrelated statistical noise. In M1 the high density of the feature maps complicates the determination of the correct consensus features. However, M2 challenges the grouping step of the tools by its large number of input maps.

**Figure 8:**
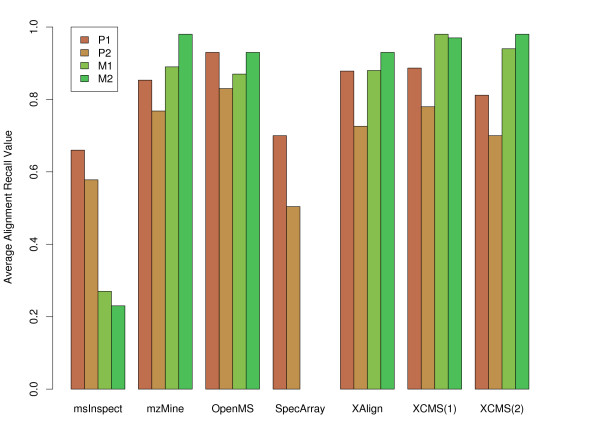
**Result Overview**. Average alignment recall values for the results on the four data sets P1, P2, M1 and M2. XCMS was evaluated without(1) and with(2) application of retention time correction. The detailed results are shown in Tables 3, 4 and 6.

Our evaluation of the tools' performance is based on alignment recall and alignment precision as defined in Section 2.4, as well as their running times. Memory consumption was not a critical resource. Since the chromatographic separation steps for the metabolomics and the proteomics data sets resemble each other, we decided to test all tools on all data sets, even though most of them were originally designed for either metabolomics or proteomics data. Figure 8 shows a summary of the results on the different data sets.

The results for the proteomics data sets P1 and P2 are shown in Tables 3, 4, and 5. We found that OpenMS performs best on P1, closely followed by XAlign, XCMS and MZmine. All four tools achieved high recall as well as high precision values on this data set. However, SpecArray and msInspect result in slightly worse recall and precision values. The evaluation on the second proteomics data set shows a similar trend, despite the overall recall and precision of all tools is reduced on this more demanding data set. OpenMS again performs best on most fractions of P2 and is closely followed by XAlign, XCMS and MZmine. SpecArray and msInspect are closely ranked after these four tools. All programs completed within two minutes on the relatively small data sets of P1 and P2.

**Table 3:** Alignment recall and precision results for the proteomics data set P1.

	msInspect	MZmine	OpenMS	SpecArray	XAlign	XCMS	
						without retention time	with correction
**fraction 00**							
Recall_Align_	0.52	0.75	**0.86**	0.61	0.82	0.72	0.62
Precision_Align_	0.38	0.81	**0.86**	0.61	0.82	0.54	0.58
**fraction 20**							
Recall_Align_	0.56	0.87	**0.92**	0.62	0.85	0.88	0.81
Precision_Align_	0.45	0.88	**0.92**	0.62	0.85	0.84	0.80
**fraction 40**							
Recall_Align_	0.63	0.87	**0.94**	0.75	0.87	0.92	0.81
Precision_Align_	0.48	0.90	**0.94**	0.75	0.87	0.85	0.80
**fraction 60**							
Recall_Align_	0.73	0.79	**0.96**	0.71	0.87	0.91	0.78
Precision_Align_	0.54	0.84	**0.96**	0.71	0.87	0.80	0.75
**fraction 80**							
Recall_Align_	0.70	0.92	**0.96**	0.74	0.90	0.94	0.89
Precision_Align_	0.57	0.94	**0.96**	0.74	0.90	0.88	0.88
**fraction 100**							
Recall_Align_	0.82	0.92	0.94	0.77	**0.96**	0.95	**0.96**
Precision_Align_	0.56	0.94	0.94	0.77	**0.96**	0.89	**0.96**

**Table 4:** Alignment recall and precision results for the proteomics data set P2.

	msInspect	MZmine	OpenMS	SpecArray	XAlign	XCMS	
						without retention time	with correction
**fraction 00**							
Recall_Align_	0.23	**0.77**	**0.77**	0.07	0.65	0.70	0.58
Precision_Align_	0.07	0.6	**0.65**	0.05	0.49	0.31	0.44
**fraction 20**							
Recall_Align_	0.67	0.87	**0.92**	0.57	0.84	0.89	0.86
Precision_Align_	0.24	0.71	**0.77**	0.42	0.70	0.55	0.66
**fraction 40**							
Recall_Align_	0.44	**0.79**	0.76	0.60	0.71	0.72	0.72
Precision_Align_	0.26	**0.76**	0.74	0.41	0.69	0.56	0.69
**fraction 80**							
Recall_Align_	0.73	0.61	**0.80**	0.65	0.58	0.64	0.49
Precision_Align_	0.34	0.56	**0.70**	0.44	0.56	0.50	0.45
**fraction 100**							
Recall_Align_	0.82	0.80	0.90	0.63	0.85	**0.95**	0.85
Precision_Align_	0.39	0.65	**0.75**	0.44	0.69	0.65	0.69

**Table 5:** Wall-clock runtime for the proteomics data sets P1 and P2 in minutes.

Data set	msInspect	MZmine	OpenMS	SpecArray	XAlign	XCMS	
						without retention time	with correction
P1	1	0.67	1.6	1.85	1.15	0.53	0.90
P2	0.75	1.22	0.36	5.19	0.29	0.33	0.49
**Total**	1.75	1.89	1.96	7.04	1.44	0.86	1.39

The results for the metabolomics data sets M1 and M2 are shown in Tables 6 and 7. Here, XCMS performs best on both data sets, and MZmine does equally well on M2, with OpenMS and XAlign not far behind. Alignment recall is much more discriminative than alignment precision, due to the penalty for breaking up a consensus feature from the ground truth. The running times were significantly different on these relatively large data sets, which contain more than 200 000 features in 24 (M1) respectively 44 (M2) feature maps. The alignments using SpecArray were canceled after 24 hours with an estimated remaining runtime of more than two weeks. SpecArray performs all pairwise map alignments and seems inapplicable to this kind of metabolomics data. In contrast, XCMS computes the alignment of the M1 and M2 in less than seven minutes. OpenMS requires 13 minutes for the determination of the metabolomics consensus maps. MZmine and XAlign both result in a high runtime of more than one hour for the quite complex metabolomics data sets.

**Table 6:** Alignment recall and precision results for the metabolomics data sets M1 and M2

Data set	msInspect	MZmine	OpenMS	SpecArray	XAlign	XCMS	
						without retention time	with correction
**M1**							
Recall_Align_	0.27	0.89	0.87	-	0.88	**0.98**	0.94
Precision_Align_	0.46	**0.74**	0.69	-	0.70	0.60	0.70
**M2**							
Recall_Align_	0.23	**0.98**	0.93	-	0.93	0.97	**0.98**
Precision_Align_	0.47	**0.84**	0.79	-	0.79	0.58	0.78

**Table 7:** Wall-clock runtime for the metabolomics data sets M1 and M2 in minutes

Data set	msInspect	MZmine	OpenMS	SpecArray	XAlign	XCMS	
						without retention time	with correction
M1	12	20	4.4	-	51	0.9	1.4
M2	24	44	8.7	-	35	5.5	5.8
**Total**	36	64	13.1	-	86	6.4	7.2

msInspect has a runtime of only half an hour, but with very low recall and precision values. We were unable to obtain good results on the data sets M1 and M2 using msInspect with parameters suggested by the optimizer as well as different values chosen manually. In most cases the automatic choice of "optimized" parameters did not lead to better alignment results than manually chosen "good" values. Furthermore, we observed that a different order of the input files leads to different results with msInspect. Placing the feature list with the highest number of features on top of the list seems to give the best results.

Another outcome of our evaluation is that it is hard to predict whether XCMS map alignment should be used with or without retention time correction, and that the characteristics of the correction need to be checked.

## 4 Discussion and conclusion

The automatic alignment of LC-MS data sets is an important step in most analysis pipelines for metabolomics and proteomics high-throughput experiments. Algorithms that perform this task efficiently and accurately have a large impact not only on basic research in biology, but also on more applied questions such as biomarker discovery and drug research in general. Due to the importance of this step and the multitude of different approaches a meaningful standard data set and a sophisticated scoring method are needed. We offer both proteomics (P1, P2) and metabolomics (M1, M2) benchmark data sets, as well as proper quality measures (Precision_Align_, Recall_Align_) and an evaluation procedure. On the basis of these data sets we have assessed the performance of six freely available alignment tools.

Perhaps surprisingly, we observed that in many cases the largest part of the *systematic *deviation of retention time in our data sets could have been corrected by a simple shift without any further scaling or non-linear warping at all. The remaining error is very similar to statistical noise, not correlated among neighboring consensus features, and further scan-wise corrections of retention time will face the risk of overfitting. This suggests that the choice of the warping function is less important than the following clustering step (i. e., the correction of the retention times of the individual features), as this will establish the actual consensus features.

The implemented methods are based on a variety of algorithmic principles with complementary strengths and weaknesses [13]. Combining them into "hybrid" approaches seems to be a promising direction for future research. However, such a project requires a long-term commitment and, if possible, several software developers are necessary. We expect to see a consolidation in the area in the future with a tendency toward open source frameworks such as Bioconductor or OpenMS.

Recently, the Association of Biomolecular Resource Facilities (ABRF) has organized a collaborative study focusing on evaluating the ability of proteomics laboratories to determine the identities of a complex mixture of proteins present in a single mass spectral *data set*, as a follow-up to an earlier study in which the actual *samples *were distributed [39]. This indicates the growing attention paid to data processing versus "wet-lab" techniques in the proteomics field. Similar competitions should be organized for all the other aspects of a typical LC-MS data processing pipeline, including the LC-MS map alignment problem. The experience from the plasma proteome project [40] has shown that it is difficult to assess the performance if many aspects change simultaneously.

We would like to encourage other MS software developers (including commercial vendors) to use our benchmark data for evaluation. Further benchmarks are also highly welcome, e. g. identical samples run at different laboratories under "identical" conditions, or even on MS equipment from different vendors. We will collect future results and contributions upon request on .

## 5 Authors' contributions

EL performed data extraction and established ground truth for the proteomics data sets, with methodology developed together with CG. RT and SN performed data extraction and established ground truth for the metabolomics data sets. EL performed parameter tuning and test runs for msInspect, SpecArray and OpenMS. RT performed parameter tuning and test runs for MZmine, XAlign and XCMS. All authors developed and decided upon the evaluation criteria. All authors read and approved the final manuscript.

## Supplementary Material

Additional file 1 The proteomics and metabolomics ground truth data sets as well as the evaluation script are available at . Click here for file
