# Supplementary material for: Critical assessment of alignment procedures for LC-MS proteomics and metabolomics measurements
Source: BMC Bioinformatics. 2008 Sep 15;9:375. doi: 10.1186/1471-2105-9-375 (PMC2570366; doi:10.1186/1471-2105-9-375)
Supplement: Additional file 1 — The proteomics and metabolomics ground truth data sets as well as the evaluation script are available at . [file 1471-2105-9-375-S1.pdf]

Additional File 1
for
Critical assessment of alignment procedures for LC-MS proteomics and metabolomics measurements
by
Eva Lange, Ralf Tautenhahn, Steffen Neumann, and Clemens Gröpl
2008-06-18

Retention time deviations of data set P1

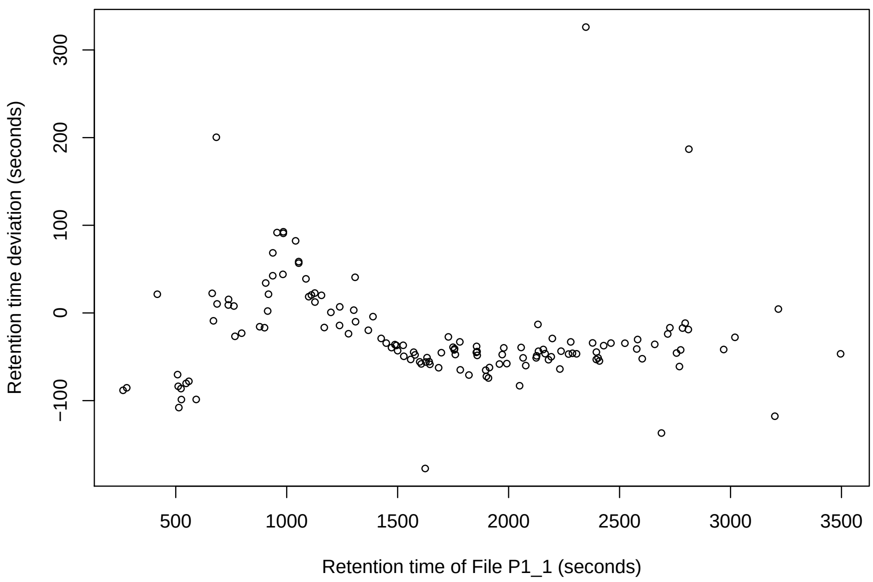

Figure 1: Ground truth: Retention time deviation of File P1_2 plotted against retention time of File P1_1 (fraction 00)

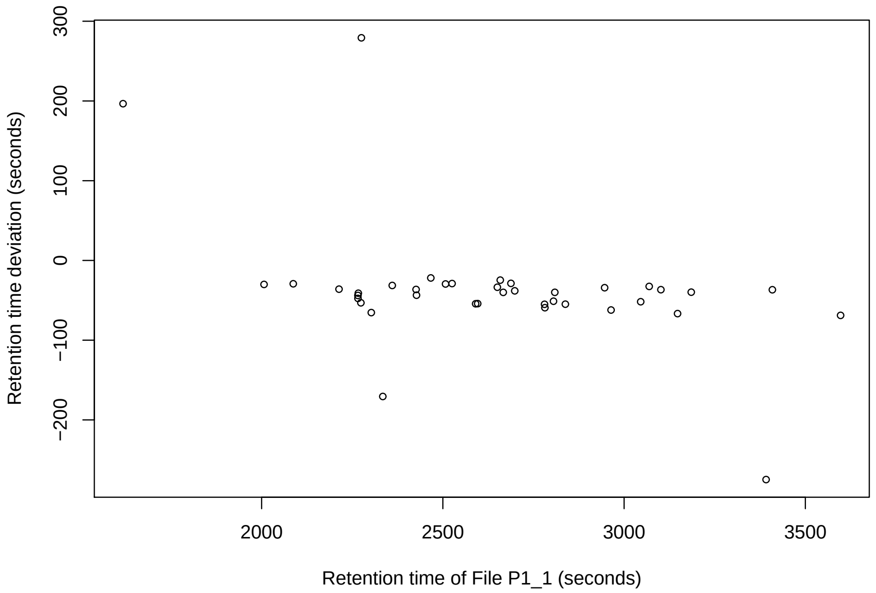

Figure 2: Ground truth: Retention time deviation of File P1_2 plotted against retention time of File P1_1 (fraction 20)

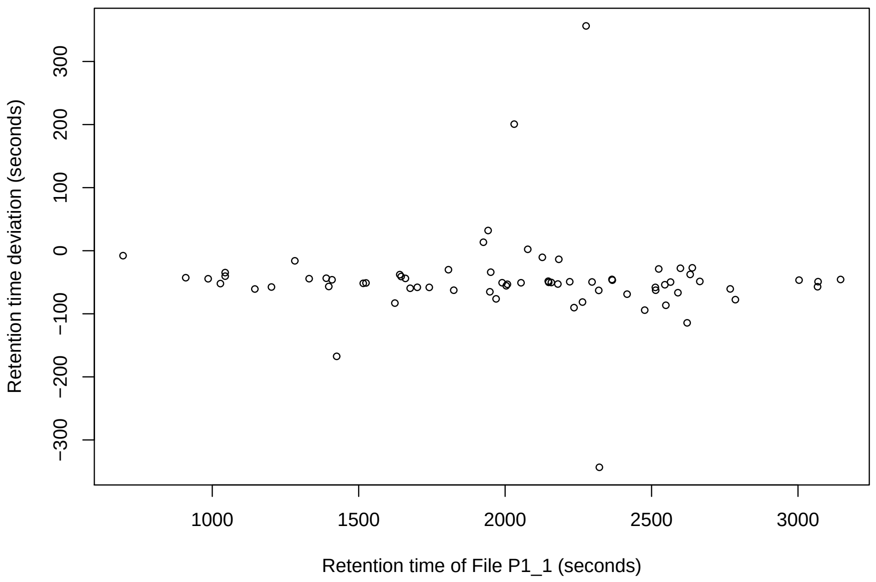

Figure 3: Ground truth: Retention time deviation of File P1_2 plotted against retention time of File P1_1 (fraction 40)

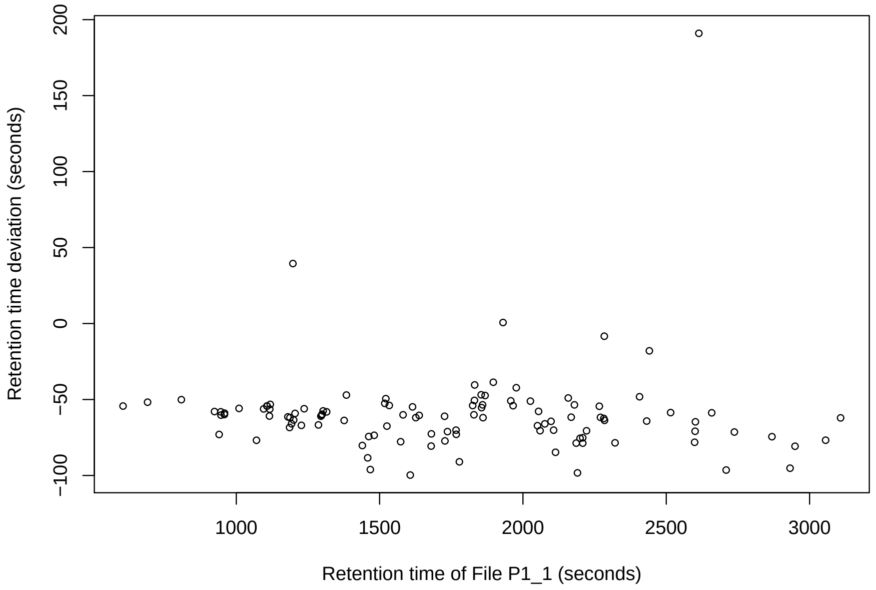

Figure 4: Ground truth: Retention time deviation of File P1_2 plotted against retention time of File P1_1 (fraction 60)

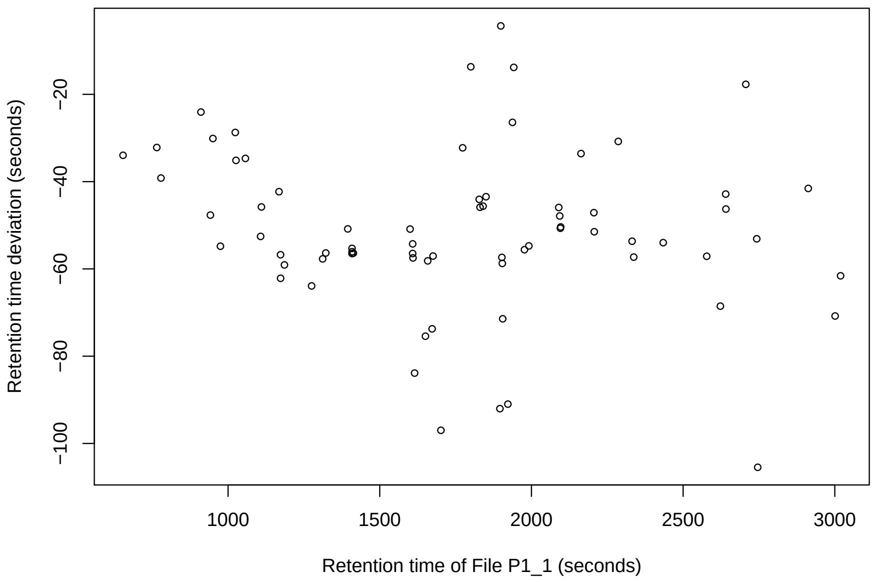

Figure 5: Ground truth: Retention time deviation of File P1_2 plotted against retention time of File P1_1 (fraction 80)

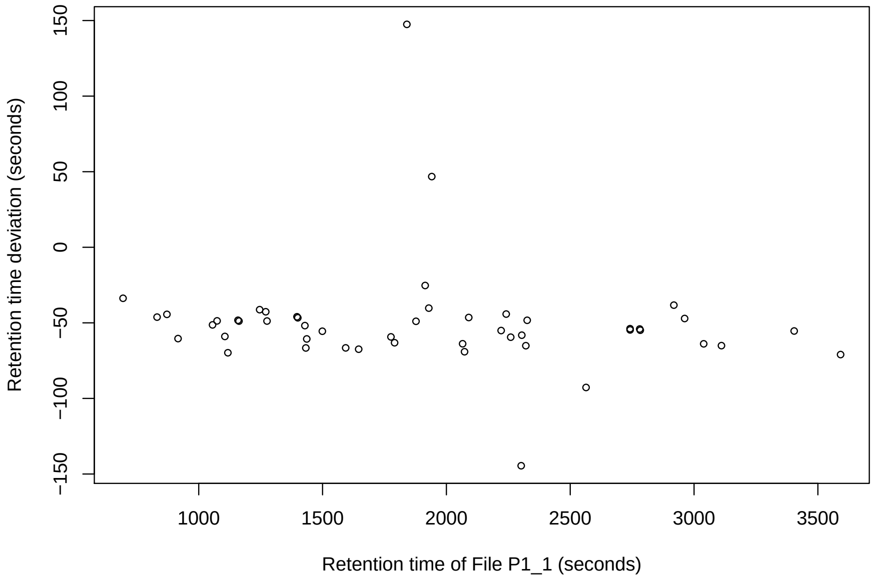

Figure 6: Ground truth: Retention time deviation of File P1_2 plotted against retention time of File P1_1 (fraction 100)

0.1 Retention time deviations of data set P2

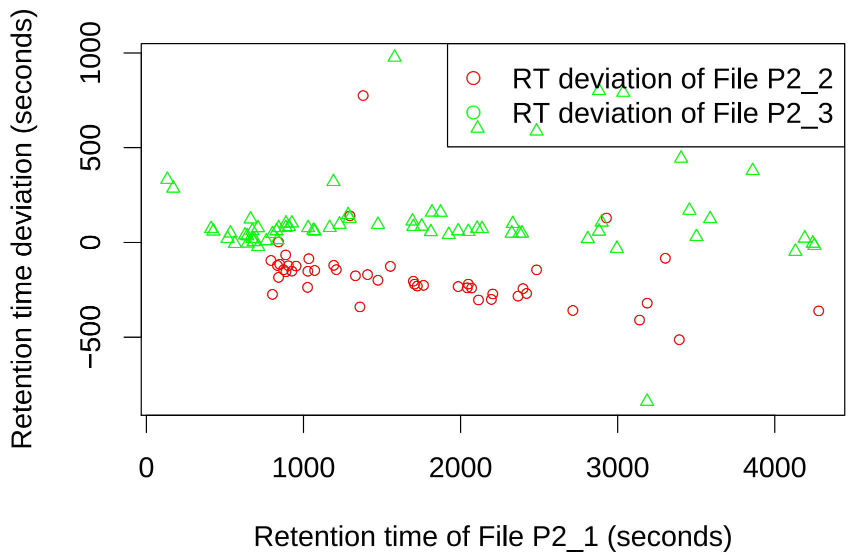

Figure 7: Ground truth: Retention time deviations of File P2_2 and P2_3 plotted against retention time of File P2_1 (fraction 00)

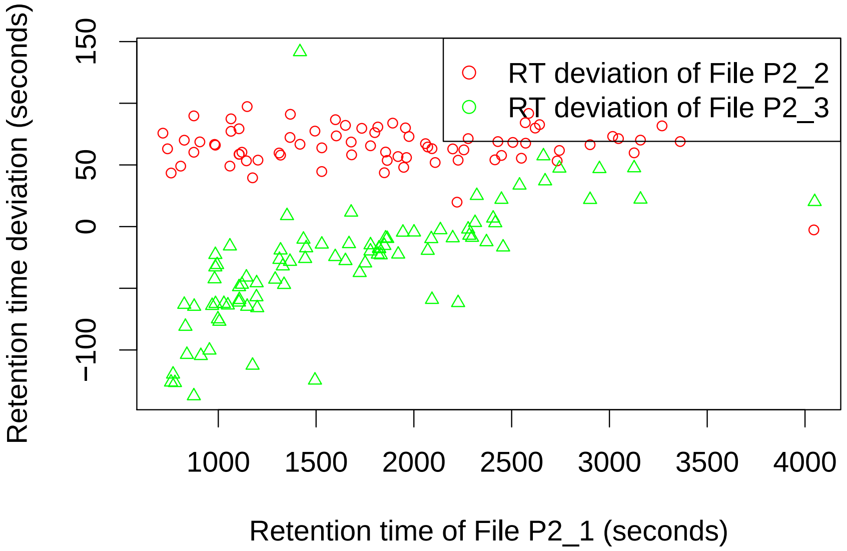

Figure 8: Ground truth: Retention time deviations of File P2_2 and P2_3 plotted against retention time of File P2_1 (fraction 20)

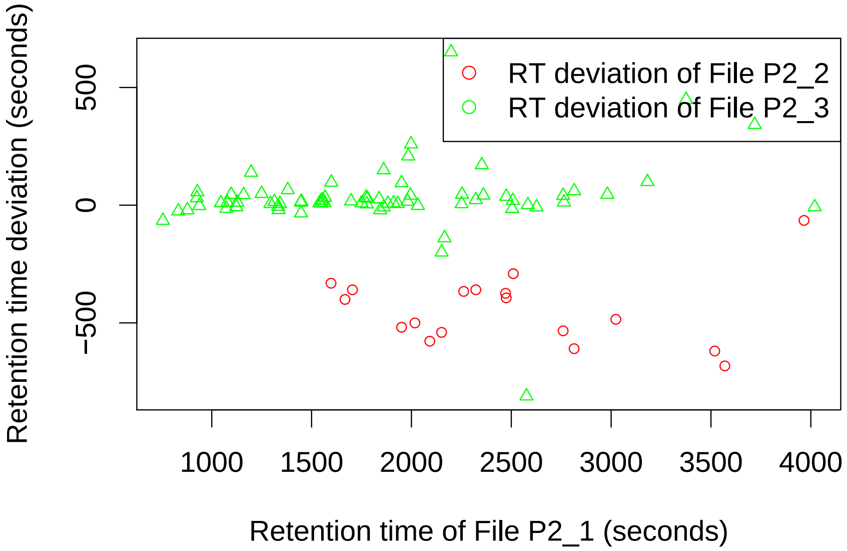

Figure 9: Ground truth: Retention time deviations of File P2_2 and P2_3 plotted against retention time of File P2_1 (fraction 40)

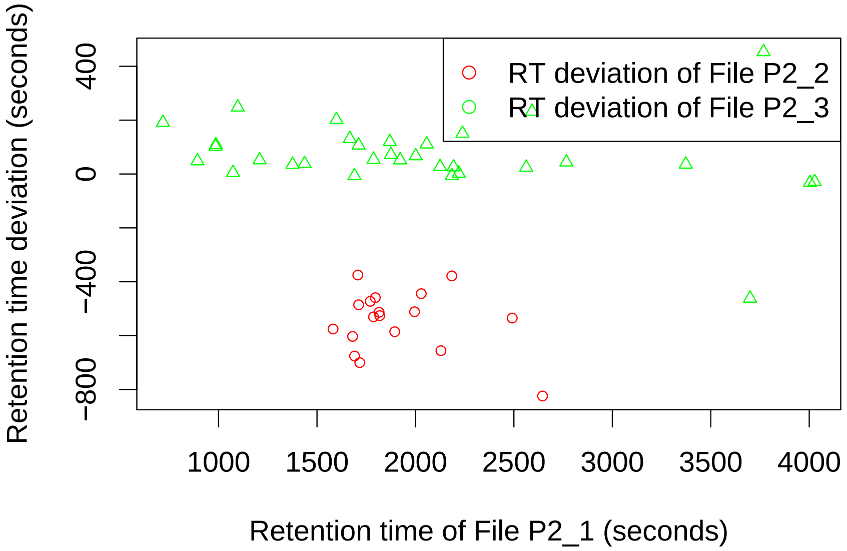

Figure 10: Ground truth: Retention time deviations of File P2_2 and P2_3 plotted against retention time of File P2_1 (fraction 80)

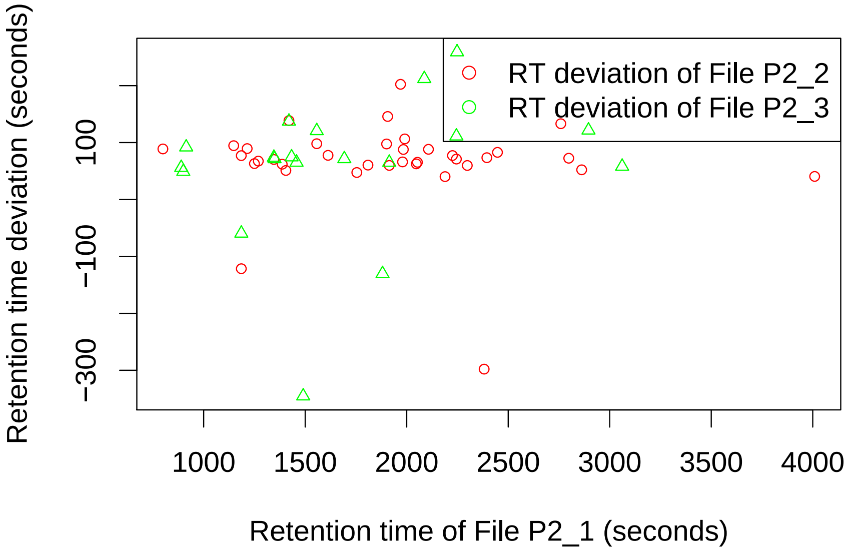

Figure 11: Ground truth: Retention time deviations of File P2_2 and P2_3 plotted against retention time of File P2_1 (fraction 100)

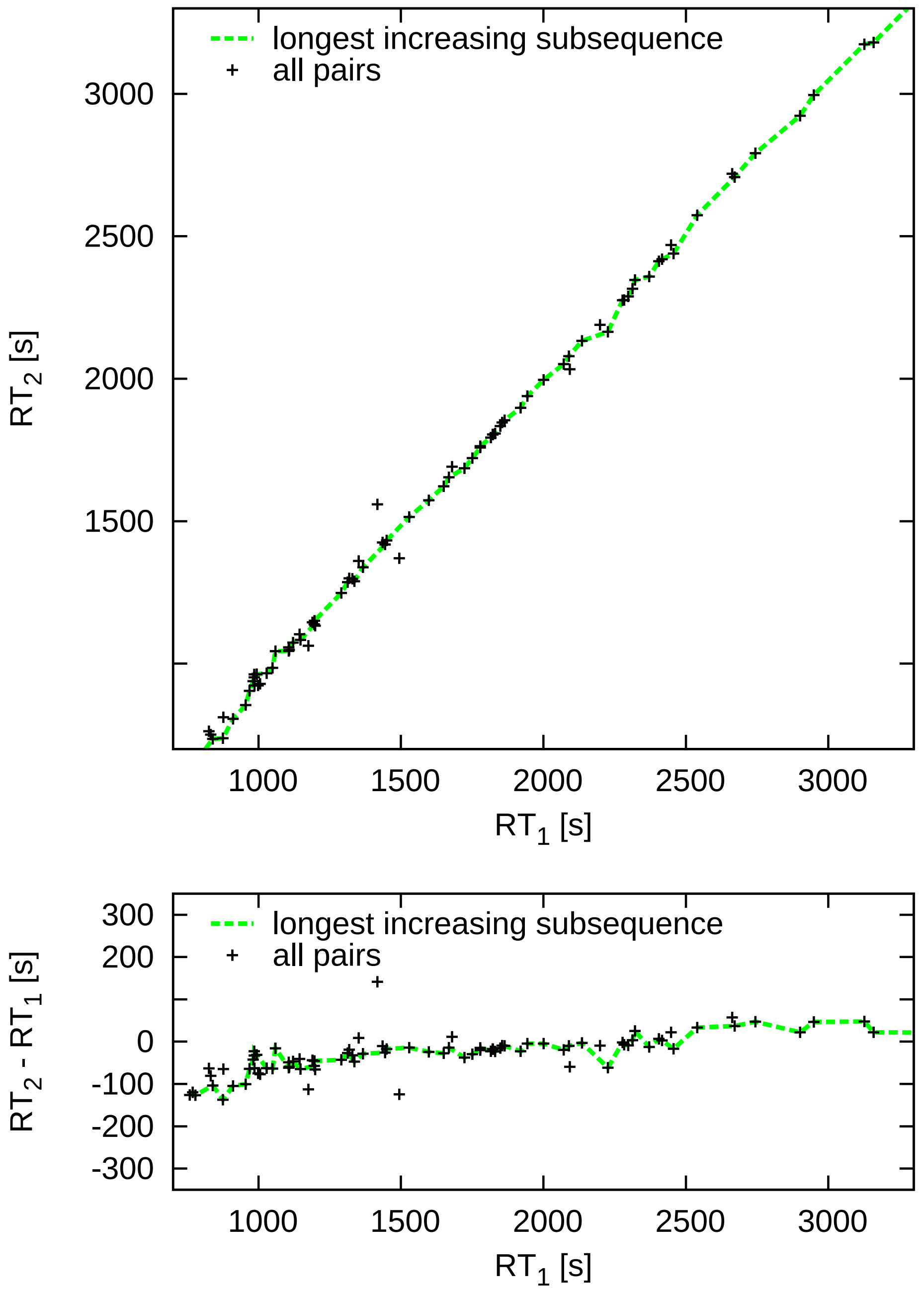

Figure 12: Ground truth: The figure shows the retention times of features in File P2_1 and File P2_3 (fraction 20). These features are matched in the ground truth. Also shown is a longest increasing subsequence that contains the maximum number of feature pairs that can be aligned without changing the elution order within each map. At most 66 of the 88 pairs can be aligned without elution order changes. The longest increasing subsequence was computed using SeqAn (http://www.seqan.de).
